# *Saccharomyces pastorianus* Residual Biomass Immobilized in a Polymer Matrix as a Biosorbent for Reactive Dye Removal: Investigations in a Dynamic System

**DOI:** 10.3390/polym16040491

**Published:** 2024-02-09

**Authors:** Daniela Suteu, Alexandra Cristina Blaga, Lacramioara Rusu, Alexandra Maria Tanasa

**Affiliations:** 1 ‘Cristofor Simionescu’ Faculty of Chemical Engineering and Environment Protection, “Gheorghe Asachi” Technical University of Iasi, 73 D. Mangeron Blvd., 700050 Iasi, Romania; danasuteu67@yahoo.com (D.S.); acblaga@tuiasi.ro (A.C.B.); alexandra_tanasa20@yahoo.com (A.M.T.); 2 Faculty of Engineering, “Vasile Alecsandri” University of Bacau, 157 Calea Mărăşeşti, 600115 Bacau, Romania

**Keywords:** biosorption, brilliant red HE-3B, fluidized bed column, natural polymer, reactive dye, removal, *Saccharomyces pastorianus* residual biomass, natural polymer

## Abstract

The use of residual microbial biomass from various industries in emerging pollutant removal strategies represents a new area of research in the field. In this case, we examined how to remove reactive dyes from an aqueous solution utilizing a biosorbent made of residual biomass from immobilized *Saccharomyces pastorianus* (*S. pastorianus*) in a polymer matrix using a dynamic system. Fluidized bed column biosorption investigations were carried out on a laboratory scale. Brilliant Red HE-3B was chosen as the target molecule. The main parameters considered for this purpose were the flow rate (4.0 mL/min; 6.1 mL/min), initial pollutant concentration (51.2 mg/L; 77.84 mg/L), and biosorbent mass (16 g; 20 g). The experimental data of the fluidized bed study were evaluated by mathematical modeling. The Yoon–Nelson, Bohart–Adams, Clark, and Yan models were investigated for an appropriate correlation with the experimental data. An acceptable fit was obtained for a flow rate of 4 mL/min, an initial pollutant concentration of 51.2 mg/L, and a biosorbent amount of 20 g. The obtained results indicate that the biosorbent can be used efficiently in a dynamic system both for the removal of the studied dye and in extended operations with a continuous flow of wastewater. As a conclusion, the investigated biocomposite material can be considered a viable biosorbent for testing in the removal of reactive dyes from aqueous environments and creates the necessary conditions for the extension of studies toward the application of these types of biosorbents in the treatment of industrial effluents loaded with organic dyes.

## 1. Introduction

As a result of global trends toward faster industrialization, several hazardous substances are discharged into the environment, which results in affecting its quality and, implicitly, the quality of people’s lives [1]. According to Chakraborty et al. [2], dyes are among dangerous and hazardous chemicals that are extensively utilized in a variety of industries, including textiles, paint, food, paper, varnishes, ink, plastics, cellulose, cosmetics, plastic, and tanning. Worldwide, around 7 × 10^7^ tons of synthetic dyes are produced annually, and the textile industries employ over 10,000 t of these colors [3]. Roughly 15% of the dyes and pigments produced by different industries are thought to end up in effluents.

The stability of aquatic ecosystems has changed as a result of the improper or untreated discharge of colored effluents into water bodies because dye obstructs light infiltration through bleaching even at very low concentrations, hinders the photosynthetic process, impedes the growth of fauna, generates micro-toxins for aquatic organisms, increases the chelating of metal ions, and increases the chemical oxygen demand [2,4]. Furthermore, dyes themselves and the byproducts of their degradation are thought to be hazardous, carcinogenic, or even mutagenic for humans. As a class of organic dyes with multiple uses, azo dyes account for 60–70% of all dye production. Because of its outstanding binding ability, beautiful color, ease of application, and color fastness, Brilliant Red HE-3B is an azo dye that is widely utilized in many different industrial sectors. Its removal is difficult due to its distinctive characteristics (e.g., aromatic ring structure, nonbiodegradability, and high heat and light stability) [2,5]. Thus, improving water quality and treating dye contamination in aquatic systems are crucial issues in environmental technologies [6,7].

Coagulation and flocculation, membrane separation (e.g., reverse osmosis and ultrafiltration), ion exchange, advanced oxidation processes (e.g., oxidation/ozonation and photocatalysis), electrochemical processes, mixed-matrix membranes and nanofiltration multi-channel membranes, biological approaches, biodegradation, adsorption, and biosorption are available treatment technologies for dye removal from wastewater [8,9]. Several authors have analyzed the advantages and limitations of these treatment technologies [10,11]. Among these technologies, biosorption is a highly efficient and cost-effective process commonly used in wastewater treatment and environmental remediation where biomass (microbial or vegetal), as a biosorbent, is used to remove environmental pollutants from a liquid or gas feed [12,13,14,15,16]. During biosorption, pollutant removal is ensured by different transport and equilibrium processes (surface and interfacial phenomena, absorption, adsorption, or ion exchange) [17,18]. Inactive microbial biomass, such as bacteria, fungi, and algae, can be used as efficient biosorbents in the removal of pollutants, especially dyes from wastewater [16,19,20,21,22,23]. The literature presents considerable research related to the removal of dyes from aqueous solutions by adsorption/biosorption, but most of them are in a static operating regime, which limits their application to industrial effluents.

Taking into account the importance of scaling up from static systems to real-time applications, the investigation of biosorption systems under dynamic conditions is of major importance [24,25,26].

In a dynamic regime, biosorption refers to the use of biosorbents in a continuous flow system, where the liquid or gas containing the pollutants is passed through an equipment that contains biological material and the pollutants are sorbed onto the biosorbent as the liquid or gas flows through the column [27,28,29]. The effectiveness of this process depends on several factors, including the type of biosorbent used, the flow rate of the liquid or gas, the concentration and composition of the pollutants, and the contact time between the pollutants and the biosorbent [12,13,14,19,24,27,28,29,30]. The advantages of biosorption in a dynamic regime include: low cost, high efficiency, and ability to remove a wide range of pollutants. However, the process requires careful monitoring and maintenance to prevent the fouling or saturation of the biological material, which can reduce its effectiveness over time [14,31]. There are several types of equipment that can be used for biosorption in a dynamic mode, including [31,32,33]:

- Packed bed systems are one of the most commonly used equipment: The biosorbent is packed into a column, and the contaminated water is passed through the column at a controlled flow rate. The pollutants are adsorbed onto the surface of the biosorbent as the water flows through the column.
- Fluidized bed systems are another type of equipment where the biosorbent is fluidized by passing air or other gas through the column, allowing the use of a high density of particles with good mixing between the phases, and requires low energy.
- Stirred reactors with membranes combine biological treatment and membrane filtration in a single system: the biosorbent is suspended in the wastewater, and a membrane filter is used to separate the clean water from the initial wastewater and biosorbent.

While packed bed reactors are a commonly used equipment for biosorption, they do have some disadvantages, including: channeling (preferential flow channels that reduces the effectiveness of the biosorption process), saturation (a saturated biosorbent cannot remove pollutants, limiting its lifespan), cost (require significant investment in terms of equipment, maintenance, and the replacement of the biological material), and scale up limitations. To reduce channeling and to improve fouling resistance, fluidized bed systems are used. They are more efficient as they allow an increased mass transfer (the biosorbent fluidization in the column creates a high surface-area-to-volume ratio and improves contact with the wastewater and are much easier to scale up, being suitable for a wide range of treatment applications [29,30,32,34].

Limited research has been conducted on efficient biosorption on a dynamic regime to remove organic pollutants from wastewater [24]. Sarikaya investigated the use of *A. campestris* (fungi) as a biosorbent for Sirius Blue K-CFN (azo dye) biosorption, obtaining an improved uptake of dye molecules with increasing packing heights with a maximum of 97.32% biosorption [19]. Costa et al. investigated the use of brewery-spent grains as a biosorbent for the removal of Reactive Blue 5 G dye in batch and continuous flow systems, obtaining a dye removal of 94.5% and optimum results in continuous systems with 4 g biosorbent and a flow rate of 2 mL/min [28]. Sayin (2022) investigated a biosorbent obtained by immobilizing *Thamnidium elegans* (fungi) on *Phragmites australis* with a biosorption yield of 96.51% and saturation biosorption capacities in the column mode of 104.58 in a Reactive Blue 49 dye solution and 70.98 mg/g in real wastewater samples [6].

Nowadays, the brewing industry uses a cold bottom-fermenting yeast called *Saccharomyces pastorianus*. With 1.82 billion hectoliters of beer produced worldwide in 2020 [35], the leftover biomass of the fungus *Saccharomyces pastorianus* (fungi) is a widely available byproduct of this process. Although this biomass is widely available and safe, its use in biosorption processes has only been described in a small number of research studies [36,37]. This makes it a potentially innovative and practical option in the biosorbents field.

Based on the important correlations previously established [38] between the properties of the biosorbent, the characteristics of the pollutant Brilliant Red HE-3B (BRed), a reactive dye, and the operational parameters that influence the biosorption process in a static regime, the biosorption of dyes in a dynamic regime can be studied in a fluidized bed column. Also, more knowledge than that provided by batch biosorption studies is required to construct a continuous effluent treatment system. To the best of our knowledge, there is no study on the removal of Brilliant Red HE-3B (reactive dye) using a biocomposite material based on microbial biomass and a natural polymer in a fluidized bed column.

Therefore, in this article, the dynamic flow biosorption conditions for the reactive Brilliant Red HE-3B (BRed) dye using *S. pastorianus*, a residual biomass from the beer industry, in an immobilized form was investigated in synthetic solutions. The influence of various parameters (flow rate, pollutant concentration, and the amount of biosorbent) was discussed. The Yoon–Nelson, Bohart–Adams, Yan, and Clark models served as the foundation for the biosorption modeling of experimental data in the dynamic regime.

The potential to expand the studies to a third stage, specifically the study of industrial effluents containing dyes, will be provided by the obtained results, which will show whether the investigated microbial biosorbent is a suitable material with sorption properties for use as a biosorbent in both static and dynamic working systems. As a result, this research will assist in establishing the operational parameters for the implementation of this type of system in an industrial setup.

## 2. Experimental Procedure

### 2.1. Materials

A local brewing company, Albrau, in Onesti, Romania, provided the residual *S. pastorianus* biomass, which was a waste of the technological process of brewing beer. The biomass was immobilized using a Buchi microencapsulator (Buchi Labortechnik AG, Flawil, Switzerland). A 1.5% low-viscosity-grade sodium alginate, made using distilled water at 70 °C and supplied by Buchi Labortechnik AG (Flawil, Switzerland), was used to immobilize the 5% biomass content. After homogenization, the suspension was dripped in a 0.5% calcium chloride solution for producing spherical beads with a diameter of 1.5 mm by using a nozzle with 750 μm diameter and in the following conditions: air pressure of 100 mbar, T = 45 °C, 500 V, and 800 Hz (for 750 μm), according to procedure described in [38].

Physico-chemical techniques (FTIR, SEM, and EDX) were used to characterize the produced polymeric composite before and after the biosorption process in a batch regime. These techniques highlighted the internal structure and the functional groups responsible for the biosorbtive properties. The procedures involved are detailed in [38]. Also, we determined the value of pH_PZC_, whose value of 5.4 suggests how chemical pollutant species can be retained on the biosorbent depending on the electrical charge of the functional groups [38].

**Figure 1:**
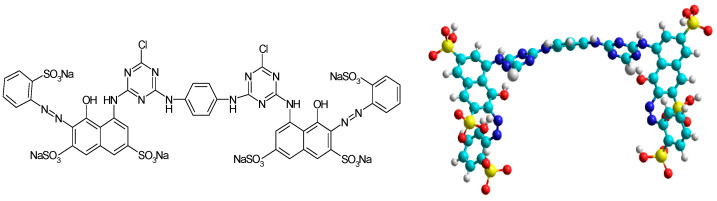
Anionic, bifunctional reactive dye Brilliant Red HE-3B (Reactive Red 120; C.I. 25810); MW = 1463 g/mol; λ _max_ = 530 nm; and concentration of the stock solution = 556 mg/L.

Dye. From the category of reactive dyes, we selected for this study the reactive dye Brilliant Red HE-3B, from Bezema Colour Solutions, Montlingen, Switzerland (also known as Reactive Red 120, C.I. 258810, BRed), considered for the experimental studies as a synthetic model for organic pollutants. Their structure and characteristics are presented in Figure 1.

Our entire research consulted numerous articles on its retention by (bio)sorption on different materials with adsorption properties, from natural or chemically synthesized to agro-industrial waste (vegetable residues, residual microbial biomass, and power plant ash), with structures based on natural (polysaccharides) and synthetic (ion exchange resins) polymers [38,39,40,41].

The working synthetic aqueous solutions were created by appropriate dilution so that they fell within the solution concentration range of 51.2–77.84 mg of dye/L using a dye stock solution.

### 2.2. Methods

#### 2.2.1. Quantitative Characterization of the Biosorption Process

The residual dye concentration in the periodically collected aqueous samples was determined spectrophotometrically by measuring the absorbance at the maximum dye wavelength of 530 nm with a Shimadzu UV-1280 UV-VIS spectrophotometer (Shimadzu Corporation, Kyoto, Japan) and using a calibration curve method (range of working concentrations chosen in the Lambert–Beer region). Using a 0.1 N HCl solution, the dye solution’s pH was adjusted in accordance with the data supplied by a portable Hanna high-accuracy KL-009(I) pH-meter (Hanna Instrument, Petite Riviere, Mauritius).

Choosing a value of 3 for the pH allowed the estimation of a biosorption capacity of 555.55 mg BRed dye/g of this microbial biosorbent at a system temperature of 25 °C [38]. The quantity of dye retained (Equation (1)) was used to assess the biosorption capabilities in the batch regime of this microbial biosorbent (q_e_).

(1) $$q_{e}=\frac{C_{0}-C_{e}}{G}\cdot V$$

where C_0_ and C_e_ are the initial and equilibrium concentrations of the dye in the solution (mg of dye/L of solution), respectively; G is the amount of the biosorbent (g): and V is the volume of the treated solution (L).

The plotting of the dye concentration in the effluent (C_t_) or the normalized dye concentration in the effluent (C_t_/C_0_) as a function of time (t) or treated volume (V) is represented by the breakthrough curve for each selected condition. This analysis was conducted with consideration of the behavior of the microbial biosorbent based on the immobilization on sodium alginate of the *S. pastorianus* biomass in the BRed dye biosorption (dynamic regime).

#### 2.2.2. Dynamic Biosorption Procedure

**Figure 2:**
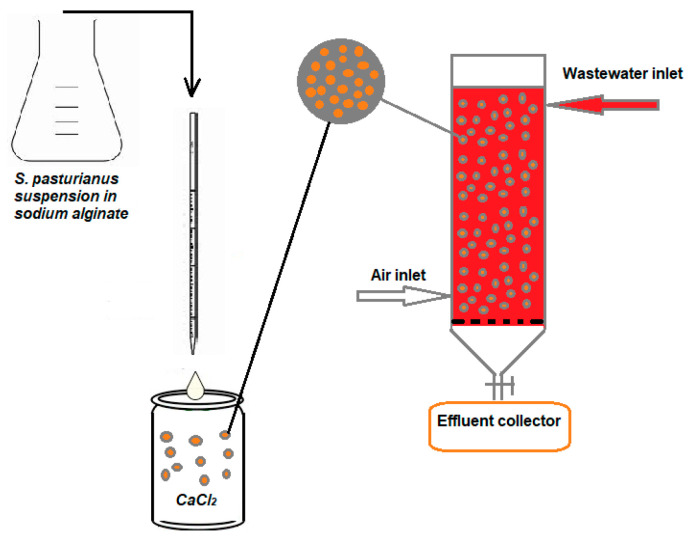
Schematic representation of biosorbent synthesis and the BRed dye biosorption process using *S. pastorianus* biomass immobilized in sodium alginate as the biosorbent.

A glass column of 15 cm in length and 3 cm in inner diameter was used for the dynamic adsorption studies. Different weights of *S. pastorianus* beads (16–20 g) were used to fill the column, resulting in a biosorbent packed bed height of (4.5–5) cm. To maintain a uniform, continuous flow, a feeding funnel equipped with a tap was used to introduce the dye solution at the top of the column with a specified concentration (51.2 mg/L and 77.84 mg/L). The dye solution was run through the column at a flow rate of 4 and 6.1 mL/min, and the effluent was collected at the bottom for additional analysis and control. Samples were taken every ten minutes (Figure 2). In order to obtain the fluidization of the biosorbent beads, an air stream was introduced intermittently every 5–10 min on the lower side of the column (with a flow rate of 0.1 L/s). The role of air during the biosorption process was only to ensure the fluidization of the bead layer (the beads having sticky surface), so that their entire specific surface comes into contact with the dye solution, thus avoiding the adhesion of the granules to each other or to the column’s glass surface.

These samples were then analyzed with a UV-VIS Digital Spectrophotometer, model Shimadzu UV-1280.

All experiments were carried out with the following operating parameters taken into account in order to assess the suggested adsorption model:

(1) Based on the best outcomes from the batch adsorption investigation, the inlet solution temperature and pH were set at 25 °C and 3, respectively [38];
(2) Two distinct flow rates (F_v1_ = 4.0 and F_v2_ = 6.1 mL/min) of the dye synthetic solutions with the same dye concentration (51.2 mg/L and 77.84 mg/L, respectively) were passed through the fluidized biosorbent bed in the column, equivalent to a biosorbent mass (m) of 20 g.
(3) Another parameter analyzed was the BRed dye initial concentration. Concentrations of 51.2 mg/L and 77.84 mg/L were selected and tested at a flow rate of 6.1 mL/min and biosorbent dose of 20 g.
(4) The third parameter analyzed was the biosorbent dose. Two doses of 16 and 20 g were tested at a flow rate of 6.1 mL/min and an initial dye concentration of 77.84 mg/L.

The experimental determinations stopped when the equilibrium was considered to be reached. This moment was determined either by obtaining a constant concentration over several time intervals or when the determination of the final dye concentration in the collected treated samples showed an equal or close value compared to the initial concentration of the dye.

### 2.3. Modeling the Biosorption Experimental Data in a Dynamic Regime

Various numerical kinetic models in their linear or nonlinear form are presented in the literature to evaluate breakthrough behavior and the effects of independent variables on a dynamic adsorption system, such as those of Thomas, Yoon–Nelson, Bohart–Adams, Clark, and Yan [24,37,42,43,44,45]. The obtained experimental data were analyzed using some of the most well-known models from the scientific literature, including the Yoon–Nelson (1), Bohart–Adams (2), Clark (3), and Yan (4) models, in order to determine the typical biosorption parameters in the dynamic biosorption system [24,42,43].

According to the Yoon–Nelson model, which has a nonlinear Equation (2), the likelihood that the biosorbate may display breakthrough behavior on a biosorbent bed is related to the biosorption rate for each biosorbate molecule decreasing [24,42,43].

(2) $$\frac{C_{t}}{C_{0}}=\frac{1}{1+e^{{(k}_{\mathrm{YN}}\cdot \tau -k_{\mathrm{YN}}\cdot t)}}$$

where C_0_ (mg/mL) and C_t_ (mg/mL) are the dye concentrations in the influent and in the effluent, respectively; k_YN_ (1/min) is the Yoon–Nelson rate constant; τ (min) is the time required for 50% breakthrough; and t (min) is the biosorption time.

With the assumption that the biosorption rate is proportionate to the quantity or concentration of biosorbate in the bulk liquid phase and to the remaining biosorption capacity of the biosorbent, the Bohart–Adams model (Equation (3)) ignores the effects of axial dispersion [24,42,44]. The Bohart–Adams model equation’s nonlinear form is as follows:

(3) $$\frac{C_{t}}{C_{0}}=\frac{e^{{(k}_{\mathrm{BA}}\cdot C_{0}\cdot t)}}{e^{{(k}_{\mathrm{BA}}\cdot C_{0}\cdot t)}+e^{{(k}_{\mathrm{BA}}\cdot N_{\mathrm{BA}}\cdot Z/v)}-1}$$

where k_BA_ is the Bohart–Adams constant (mL/(mg·min)); C_0_ (mg/mL) and C_t_ (mg/mL) are the dye concentrations in the influent and in the effluent, respectively; N_BA_ (mg/mL) is the biosorbent adsorption capacity for the unit volume of the bed; Z (m) is the bed depth; v (m/min) is the feed velocity; and t (min) is the biosorption time.

The Clark model (Equation (4)) is based on the mass transfer phenomenon combined with the Freundlich isotherm. The following hypotheses are proposed in this model: the mass transfer in the outer film is the process’s limiting step; all of the adsorbate is removed at the column’s end; and the mass transfer zone’s shape is constant [24,42,44].

(4) $${\frac{C_{t}}{C_{0}}}^{,}={\left(\frac{1}{1+A_{\mathrm{ck}}\cdot e^{(-r_{\mathrm{ck}}\cdot t)}}\right)}^{\left(\frac{1}{n_{\mathrm{fr}}-1}\right)}$$

where C_0_ (mg/mL) and C_t_ (mg/mL) are the dye concentrations in the influent and in the effluent, respectively; A_ck_ (dimensionless) and r_ck_ (1/min) are Clark model parameters; n_fr_ is the Freundlich parameter; and t (min) is the biosorption time.

Yan (2001) suggested a model (Equation (5)) with the main goal of minimizing the mathematical adjustment error of the Thomas model. The model fitted the adsorption column curve using an empirical approach [37,42]. Its nonlinear form is represented by Equation (5):

(5) $${\frac{C_{t}}{C_{0}}}^{,}=1-\frac{1}{{\left(1+e^{{(C}_{0}\cdot Q\cdot {t/q}_{Y}\cdot m)}\right)}^{k_{Y}}}$$

where C_0_ (mg/mL) and C_t_ (mg/mL) are the dye concentrations in the influent and in the effluent, respectively; Q is the flow rate (mL/min); t (min) is the biosorption time; m (g) is the biosorbent mass; k_Y_ (dimensionless) is the Yan constant rate; and q_Y_ (mg/g) is the biosorbent maximum adsorption capacity.

The linear regression method was used to compare the models and the experimental data, and for the best agreement between the calculated model values and the experimental values, the linear regression coefficients (R^2^) must be as close to the unity as possible.

## 3. Results and Discussion

These are the results of the second stage of the in-depth study on the retention of persistent organic pollutants (i.e., textile dyes) from aqueous media on biosorbents based on residual microbial biomass immobilized in polymer matrices. In the first stage of the study conducted under a batch regime, the operating conditions (T, initial pH, initial concentration of the dye solution, and biosorbent dose), the characterization of the biosorbent (using FTIR and SEM_EDX methods), and the determination of the retention mechanism (by modeling the process based on the Langmuir, Freundlich, and Dubinin–Radushkevich models) were investigated. Thus, the biosorption process of the reactive dye BRed was spontaneous and exothermic nature. The retention mechanism was based on physical bonds (the energy value of the Dubinin–Radushkevich model was 8–9 KJ/mol) [38].

**Figure 3:**
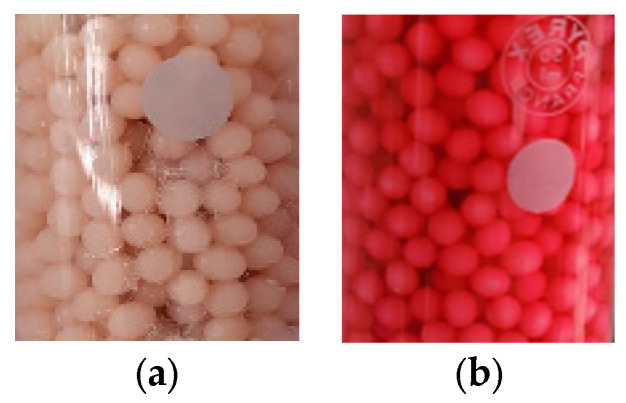
Images of the used biosorbent before (**a**) and after the biosorption (**b**) of the reactive dye BRed from the aqueous solution in a fluidized bed column.

Considering the fact that the detailed characterization of the biosorbent before and after biosorption was presented in the paper for biosorption in a static regime [38], in this paper, we briefly present some data and images. The biosorbent beads (Figure 3) have a regular and spherical shape with dimensions of 1.50 mm ± 0.13 mm.

Among the parameters studied in the static operating regime, the pH and temperature are important for the dynamic operating regime. Therefore, considering that the obtained results show that the best efficiency is at a pH of the initial dye solution of 3 (pH_PZC_ = 5.4 [38]) and a temperature of 25 °C, we considered the values of these parameters for the study in the continuous operating mode [38].

### 3.1. Factors That Influence the Biosorption Process in the Dynamic Regime

**Figure 4:**
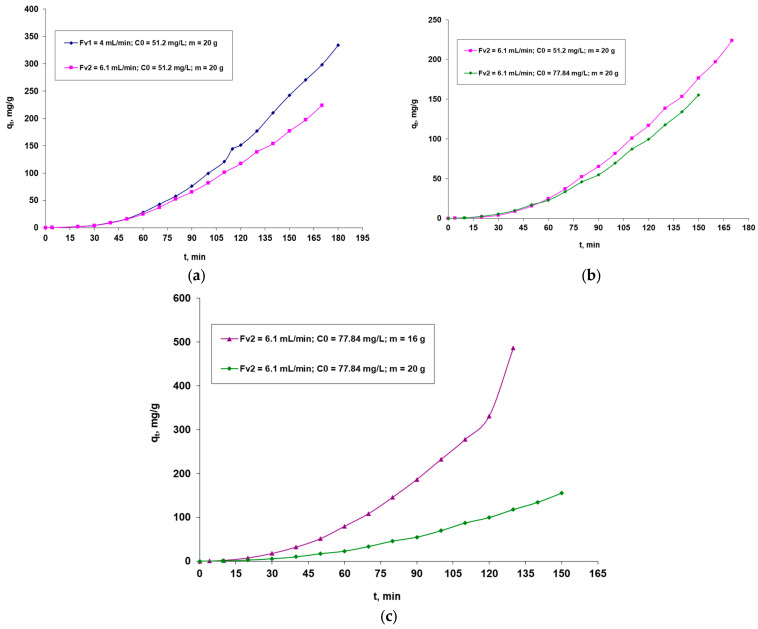
Factors that influence the biosorption of the Brilliant Red HE-3B reactive dye onto the biosorbent based on the immobilized residual biomass of *S. pastorianus* in sodium alginate in a dynamic regime (C_t_—dye concentration at time t; mg/L is directly proportional to the biosorption capacity, q_t_ mg/g): (**a**)—influence of initial flow rate; (**b**)—influence of initial dye concentration; (**c**)—influence of the amount of biosorbent.

The studied biosorption process is influenced by the factors that directly affect the performance of the system, especially the biosorption capacity. It is concerned with the flow rate through the column of the dye solution, the initial concentration of the dye, and the mass of the biosorbent in the column. In Figure 4, the outcomes are displayed graphically.

Analyzing Figure 4, it can be seen that:

(i) For the same initial dye concentration and the same amount of biosorbent in the fluidized bed, the biosorption capacity (q_t_, mg/g) is positively influenced by the passage of the solution through the column at a lower flow rate (F_v_, mL/min), as this ensures a longer contact between it and the biosorbent layer, which favors a better retention of the high-molecular-weight dye;
(ii) For the same flow rate of the solution (F_v_, mL/min) through the column and the same amount of biosorbent in the fluidized layer, it is observed that the retention of the dye is better in the case of the solution with a lower concentration of the dye. This behavior can be explained by the fact that the active centers of the biosorbent become more accessible with a larger amount of dye, since the molecules are more mobile at a lower concentration and can diffuse more easily to the surface of the biosorbent.
(iii) For the same initial concentration of dye and the same flow rate of the solution flowing through the column, an improved biosorption of the dye is observed in the case of a lower biosorbent dose. This behavior can be explained by taking into account the large size of the adsorbate molecules and the possible steric hindrances that may occur during their dissolution. A lower dosage of the biosorbent leads to less compaction of the layer, which facilitates the diffusion of the bulky dye molecules to the active sites.

### 3.2. Breakthrough Curves

**Figure 5:**
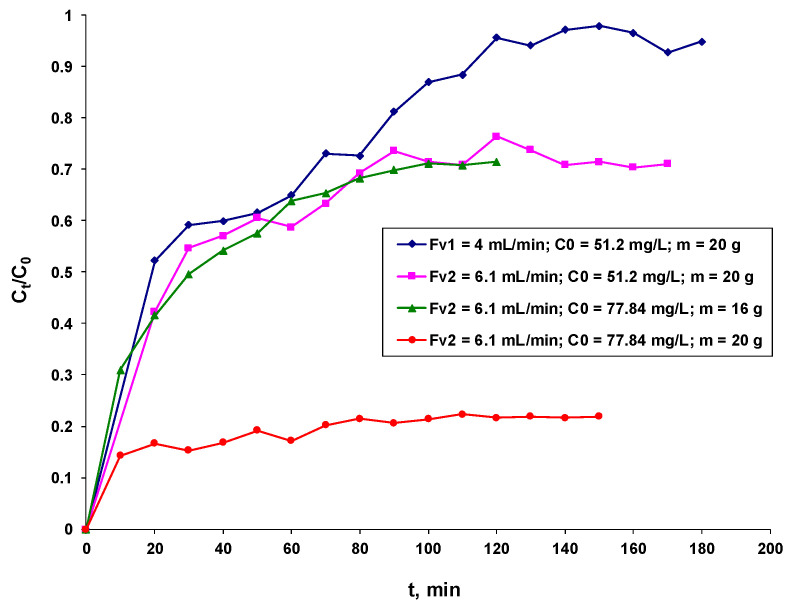
Effect of the operational parameters on the breakthrough curves for the biosorption of the BRed dye on the biosorbent based on the residual *S. pastorianus* biomass immobilized in sodium alginate.

Figure 5 shows the breakthrough curves for BRed dye retention on a column packed with the synthesized biosorbent under continuous fluidized bed operating conditions.

The breakthrough curves are expressed as the ratio of the dye concentrations at a given time to the initial (C_t_/C_o_, mg dye/L of solution) and the adsorption time profile (t, min) [42,43].

Its structure provides some basic details about the biosorption process under study as well as how the dye loads into the continuous column.

As illustrated in Figure 5, the experiment performed with a BRed dye solution with an initial concentration of 51.2 mg/L and a flow rate of 4 mL/min demonstrates that the interactions between the biosorbent bed and the dye molecule are favorable at a lower flow rate and lower concentration.

Comparable outcomes have been reported in other published studies on the removal of various dyes from aqueous solutions in a continuous system.

Alardhi et al. [44] studied the adsorption of the methyl green dye pollutant from an aqueous solution using mesoporous materials MCM-41 in a fixed bed column and arrived to the conclusion that the highest bed capacity of 20.97 mg/g was obtained at a lower flow rate (0.8 mL/min). Rouf and Nagapadma [43] investigated the removal of Brilliant Black BN azo dye from an aqueous solution by adsorption in a fixed bed column using chitosan beads impregnated with a cationic surfactant Cetyl Trimethyl Ammonium Bromide. Their experiments revealed that the dye removal percentage increased with the decrease in the flow rate and increase in the bed height. At a flow rate of 0.8 mL/min, a bed height of 8 cm, and an initial dye concentration of 100 ppm, the maximum bed capacity of 6.80 mg was achieved. Rivera-Arenas et al. [45] evaluated the use of organoclay/alginate hydrogels in the removal of the anionic dye Acid Yellow 23 in a dynamic system. The investigations conducted in fixed bed columns revealed that, as the bed height increased, the breakthrough and exhaustion periods became longer. However, these times decreased as the flow rate and initial dye concentration increased.

### 3.3. Modeling the Dynamic Biosorption Process

Using a few models from the literature, the experimental data were processed to identify the typical characteristics of the dynamic biosorption system: Yoon–Nelson (Equation (2)), Bohart–Adams (Equation (3)), Clark (Equation (4)), and Yan (Equation (5)) models [16,37,46,47,48,49].

The plotting of the experimental data was conducted in the dynamic regime, taking into account the nonlinear form of the chosen models for the biosorption investigation. An assessment of the fluidized bed system’s effectiveness was determined by assessing its features.

**Figure 6:**
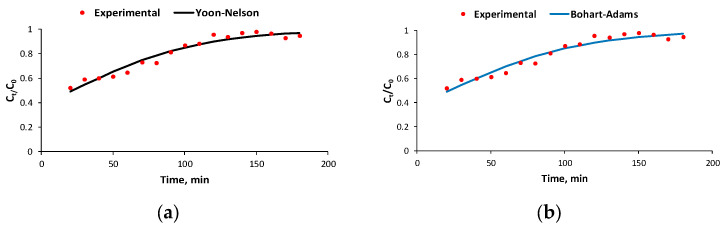

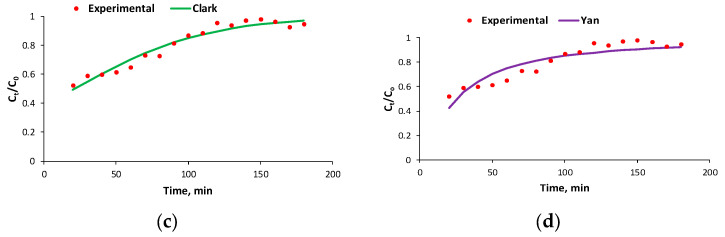
Experimental and predicted breakthrough curves using the Yoon–Nelson (**a**), Bohart–Adams (**b**), Clark (**c**), and Yan (**d**) models for the biosorption process of BRed dye with the *S. pastorianus* biomass-based biocomposite material in the dynamic system at an inlet concentration of 51.2 mg/L, flow rate of 4 mL/min, and biosorbent amount of 20 g.

The results obtained after processing the experimental data based on the nonlinear equations of the selected models led to the following conclusions:

- When the flow rate is 4 mL/min, C_0_ = 51.2 mg/L, and m = 20 g, the data match the Yoon–Nelson, Bohart–Adams, and Clark models (Table 1, Figure 6) with the same value for the correlation coefficient R^2^;
- When the flow rate is 6.1 mL/min, the only matched model is that of Yan with values for R^2^ in the range of 0.87005–0.992838. The best value for the regression coefficient (0.992838) was obtained for C_0_ = 77.84 mg/L and biosorbent mass = 16 g (Table 2)

The best results were obtained at a lower flow rate, as indicated by Table 2 and Figure 5. Consequently, the recorded experimental data were satisfactorily matched by three of the four applied mathematical models (Yoon–Nelson, Bohart–Adams, and Clark), with an accuracy degree of 0.9522.

The kinetic models of Bohart–Adams, Yoon–Nelson, Clark, and Yan have been evaluated by a number of researchers [37,43,44,45,46,47] to describe the adsorption processes in a dynamic system. The results obtained by Das et al. (2023) [46] suggested that the biosorption performance of fluoride using alginate nanocellulose beads in a fluidized bed system can be adequately predicted by applying the nonlinear forms of the Yoon–Nelson and Thomas models. de Oliveira et al. (2023) [49], when evaluating the efficiency and applicability of fixed bed columns for the adsorption of tetracycline on granular activated carbon, concluded that the Yan model was suitable for elucidating the column mechanisms, the process being controlled by surface mass transfer. Amador et al. evaluated the kinetic models of Clark, Yan, Yoon–Nelson, Gompertz, and Log–Gompertz by the Bayesian technique to represent the experimental data of the breakthrough curve from the adsorption of paracetamol in a fixed bed column filled with activated carbon, concluded that the Yan model was the most suitable [50].

Taking into account the previously presented aspects, we can note that the studied models can be used to estimate the biosorption of BRed dye on the synthesized biosorbent.

### 3.4. Dye-Loaded Biosorbent Regeneration Study

Desorption involves working in a basic environment, which results in the irreversible deterioration of the polymer matrix as well as the distortion of the granule structure caused by swelling or breaking. In this case, this type of biosorbent cannot be regenerated, but it can be recovered using other recognized ways, such as composting or anaerobic digestion processes, and used as soil amendment [51,52].

## 4. Conclusions

The efficiency of a biosorbent based on *S. pastorianus* residual biomass immobilized in calcium alginate to remove BRed dye from aqueous solutions in a dynamic system was investigated. The study examined the impact of various column functional parameters on the biosorption process, including the flow rate, BRed dye initial concentration, and biosorbent mass.

The findings show that, for the same initial dye concentration and biosorbent mass, in a fluidized layer, biosorption is positively impacted by the solution passing through the column at a slower speed (F_v_ = 4 mL/min), as this ensures a longer contact between the phases, which improves dye retention.

The Yoon–Nelson, Bohart–Adams, Clark, and Yan mathematical models were evaluated to predict and characterize the BRed dye biosorption process. The Bohart–Adams, Yoon–Nelson, and Clark models were found to fit the experimental data satisfactorily for a flow rate of 4 mL/min, an initial pollutant concentration of 51.2 mg/L, and a biosorbent quantity of 20 g.

The results suggest that the *S. pastorianus* residual biomass/alginate biocomposite material can be applied as a suitable and effective biosorbent in dye removal processes from aqueous media both in static and dynamic operating regimes. Future research investigating both textile industry effluents and other types of effluents containing dyes is required in order to advance the process to a higher technological level.

Finally, the continuous biosorption system ensures a better adsorbent–adsorbate interaction, which improves the performance and applicability of the process.

## Figures and Tables

**Table 1:** Dynamic modeling of the biosorption process with the BRed dye flow rate of 4 mL/min (C_0_ = 51.2 mg/L; biosorbent mass = 20 g).

Model	*k_BA_*	*N_BA_*	*k_YN_*	*τ*	*r_ck_*	*A_ck_*	*k_Y_*	*q_Y_*	*R* ^2^
Bohart–Adams	0.430281	−0.65857	-	-	-	-	-	-	0.952256
Yoon–Nelson	-	-	0.02203	21.51073	-	-	-	-	0.952257
Clark	-	-	-	-	0.02203	1.606224	-	-	0.952257
Yan	-	-	-	-	-	-	1.263763	0.258917	0.842638

**Table 2:** Dynamic modeling of the biosorption process with the BRed dye flow rate of 6.1 mL/min (C_0_ = 51.2 mg/L; biosorbent mass = 20 g).

Model	*k_BA_*	*N_BA_*	*k_YN_*	*τ*	*r_ck_*	*A_ck_*	*k_Y_*	*q_Y_*	*R* ^2^
Bohart–Adams	0.19403	−1.29097							0.889542
Yoon–Nelson			0.015103	36.98367					0.889542
Clark					0.015103	1.74818			0.889542
Yan							0.736814	0.908448	0.992838

## Data Availability

Data are contained within the article.
